# Children with and without reading difficulty value robot reading companions that are smart, supportive, and personalised

**DOI:** 10.1038/s41598-025-15341-w

**Published:** 2025-10-01

**Authors:** Ryssa Moffat, Hannah Cahill, Emily S. Cross, Nathan Caruana

**Affiliations:** 1 Social Brain Sciences Lab, ETH Zurich, Zurich, Switzerland; 2 School of Psychological Sciences, Maquarie University, Sydney, Australia; 3 College of Education, Psychology and Social Work, Flinders University, Adelaide, Australia; 4 Flinders Institute for Mental Health and Wellbeing, Flinders University, Adelaide, Australia

**Keywords:** Child–robot interaction, Co-design, Social robots, Reading, Education, Human behaviour, Computer science

## Abstract

As social robots are being increasingly deployed in educational settings, it is critically important to understand which design features make robots most likely to be accepted by children and to deliver benefits in education contexts. To deepen our understanding of young readers’ expectations, needs, and desires for a robot that supports reading, we conducted co-design sessions followed by a semi-structured interview with children aged 5–9 years (30 children with typical reading and a case series of five children with poor reading, of which four were enroled in reading interventions at the time the study was conducted and were regularly attending one-on-one reading remediation sessions). We applied a mixed-method approach including thematic analysis of interview data using a reflexive and inductive approach. The children in this study designed robots that could deliver reading-specific support alongside more general emotional support. Key functional features and capabilities included prosocial behaviours (smiling, play, and conversation), breadth of knowledge that assumed access to information about many topics, including core academic skills (reading and mathematics). Key aesthetic features included colourful, compact, and customisable designs. These findings have implications for the design and implementation of social robots to support reading and suggest that children are very much open to interacting with reading robot companions.

**Supplementary Information:**

The online version contains supplementary material available at 10.1038/s41598-025-15341-w.

## Introduction

Robots can now be found providing support to humans across many settings including hospitality, healthcare, and education. Over the past 15 years, the potential use of robots in education has been explored with increasing intensity^1,2^, as a tool to improve learning outcomes and engagement via agents that are intelligent, scalable and can assume different social roles in dyadic learning scenarios (i.e., novice, peer, tutor). The growing body of literature is disorganised, in that the robots evaluated in education studies are often selected because they are readily availability, and not necessarily because they possess features believed to be important for delivering the intended support. Indeed, almost no studies have specifically interrogated the aesthetic or functional aspects of robot design that are important for different education applications with different target learners. As a result, it remains unclear which robot features, in terms of design and function, optimise robots for success in education contexts^3^.

In the following sections, we summarise important findings from studies that have evaluated existing reading robots in educational environments, studies that have examined children’s perspectives on robotic reading companions, and finally, studies that have explored children’s suggestions regarding robot features suited for education and reading-specific contexts. Occasional excursions into studies examining children’s perceptions of general-purpose social robots are included for contextual enrichment.

### Existing robots employed to improve reading outcomes

A primary stream of research on educational social robots has focussed on learning outcomes, including literacy and math skills, as well as on learning engagement more generally^4^. Studies in this stream have typically taken a single commercially available robot into a classroom, where specific learning outcomes are assessed. That is, off-the-shelf robots are deployed in assistive roles with little consideration granted to children’s perceptions of and reactions to the robot’s capacities and appearance. Robot selection for these studies is often opportunisitic (such as using a NAO robot because it is a readily available robot model) rather than guided by the robot features that are best suited to the users’ needs or the learning objective in question. This is problematic in that it can lead to the overgeneralisation of findings and underestimation of robot multiplicity (i.e., positive outcomes with one robot may not generalise to one of the many other available robots). As such, rather than simply examining whether specific robots are useful, it is critical to improve our understanding of *which robot features* are useful in specific contexts, such as education. This requires a systematic scientific approach to robot design, an approach that has largely been lacking from many aspects of human–robot interaction research.

Despite this dearth of clarity regarding the specific features that promote positive experiences with robots, numerous studies have nonetheless placed robots in educational settings to observe whether robots could enhance learning outcomes (see review papers^1,5,6)^. Such studies have focussed on numeracy and literacy skills, as these skills are foundational for children’s later academic success^7,8^.

Reading-specific studies report enhancements on reading measures following reading-focussed activities with social robots^9–12^. Gordon and Breazeal^9^ measured vocabulary using a story-creation game, where children aged 4–8 years tapped written words on a tablet to teach the DragonBot [zoomorphic robot created by the lab] spoken words that the robot did not yet know. New words were introduced either randomly or according to an algorithm based on age and reading level. Increased word knowledge was observed for both random and algorithm-based conditions. Hsiao et al.^10^ compared literacy skills, including word reading, before and after children aged 2–3 years played a literacy game with iRobiQ [humanoid, robot with a screen; commercially available at the time of the study] or on a tablet twice a week for two months. The authors found that children who played with iRobiQ showed greater improvements on most aspects of the pre-post literacy assessment and propose that time spent gazing at and touching robot’s screen may have played a role in the observed improvements. Hyun et al.^11^ compared 4–5 year old children’s word recognition before and after word-learning games with iRobiQ or on a laptop, and found a significantly greater enhancement in word recognition following engagement with the robot than the laptop. Most recently, Yadollahi et al.^12^ had NAO [humanoid robot; currently commercially available] read to children aged 6–7 years and make mistakes that children were meant to notice and correct. The researchers compared children’s recognition of mistakes when NAO pointed or did not point at the text. They found that pointing enhanced children’s error recognition in some cases, while potentially introducing a distraction for less proficient readers.

While these finding demonstrate that reading robot companions hold some promise (a fact that should be established via appropriately powered replications), the findings are strongly limited by the lack of generalisability to the many other social robots that are commercially available and offer different features. This multiplicity problem is amplified by the fact that the robots differ not only in their appearance and function, but also in their social role (see review by Belpaeme and colleagues^1^, with studies positioning the robot as a peer^13^, tutor^9^, or novice^12)^. One avenue to address this challenge is to expose children to a variety of different robots that each differ by a single feature – though this is difficult to achieve. Alternatively, children’s qualitative perceptions of existing and imagined robot reading companions can inform our understanding of the influence and importance of individual features in a particular context of interest (e.g., education or literacy support).

### Children’s perceptions of reading robot features

A second stream of research on robots in educational settings takes users’ perspectives on experiences with robots into account^14^ by allowing children and families to interact with assistive robots (e.g., for reading) and subsequently exploring the users’ qualitative insights^15–18^. Such studies shed light on the strengths and weaknesses of existing robots in their intended settings.

Michaelis and Mutlu^16^ compared a 13-day robot-facilitated reading program to a paper-based program involving reading logs. The authors positioned Minnie [a commercially available, 3D printable humanoid robot commercially known as MAKI] in homes, where children aged 10–12 years read aloud to Minnie. Minnie tracked children’s page in the book, made comments on plot points, and expressed general enjoyment of the reading. Both programs led to similar reading engagement. Children who had read to Minnie believed that their comprehension improved and showed greater reading motivation at the end of the program.

In a subsequent study, Michaelis and Mutlu^17^ had students (ages 10–12 years) read science textbooks with the Minnie robot in two conditions. In one, Minnie exhibited ‘socially adept’ behaviours including maintaining and averting gaze when appropriate, remembering previous interactions, and making personalised recommendations. In the control condition, the robot did not exhibit socially adept behaviours. Minnie’s socially adept behaviour, relative to the control condition, was associated with greater ratings of social connectedness, robot socialness, mutual liking, attractiveness, as well as situational interest, but no differences relating to intelligence or human-likeness. In the interviews, presented separately^18^, children expressed that reading aloud to the robot allowed the robot to guide their attention and that they slowed their reading, thereby gaining a deeper understanding of the material.

Our research group recently explored children’s expectations, preferences, and desires for reading robot companions^15^. We found that both form and function to play distinct roles in children’s perceptions and engagement experiences when reading. In a mixed-methods study, children aged 5–12 years chose one robot from a selection of existing robots (Cozmo [mechanoid], NAO [humanoid], MiRo [zoomorphic]) to whom they read a short age-appropriate book. Beyond belonging to different categories, these three robots have very different functionalities: NAO can speak conversationally and move in humanlike ways; MiRo offers tactile responsiveness and emits burble-like sounds; Cozmo shows expressive emotions via a screen-face and non-verbal sounds. In this previous study, the selected robot was controlled by an out-of-sight experimenter and made appropriate emotional responses, involving vocalisation and small movements, to plot points. Subsequently, children engaged in a semistructured interview with the experimenter about the reading experience. Most children selected the humanoid robot, followed by the mechanoid, then zoomorphic robot. The qualitative analysis revealed that children enjoyed reading with a robot, because it was ‘cool’ and ‘fun’, but also because children found the robot to offer a supportive and calming presence. Children reported finding the robot both engaging and distracting and perceived a robot’s capacity to speak as a sign of intelligence.

These studies drive home the impact of feature differences on children’s perceptions of various robots while also highlighting children’s general enthusiasm for reading with robotic reading companions, which may last more than 12–14 weeks^19^. However, the studies in this section also share an important limitation: When existing robots are employed, they have a pre-set appearance and are programmed with specific responses that shape children’s perceptions. Taking examples from Caruana et al.^15^, NAO’s fixed gaze was interpreted variously as comforting or concerning. Moreover, when Cozmo (the mechanoid robot) made an emotional response, it would often roll over the corner of the child’s book, which some children described as distracting. One way to overcome this limitation is to avoid priming children with robot exemplars and to openly probe their expectations and desires relating to robotic reading companions using design activities.

### Children’s reading robot designs

A third stream of research on educational robots examines the unique insights into robot features that are gained by involving the users (e.g., children learning to read) in a design process^20,21^. This co-design approach typically involves drawing^22^, crafting and model-building^23,24^, and storytelling^21,25^ in combination with questionnaires or interviews.

Before diving into co-designed reading robot studies, we take a brief excursion into children’s understanding of robotic systems and the affective attributes that children ascribe to robots of varying forms. Overarchingly, children show a strong tendency to anthropomorphise robots and interact with them as if they were humans, possessing mental and social capacities^26–29^. Further evidence suggests that younger children view robots as toys with greater intelligence than themselves, whereas older children view robots as humans with less intelligence than themselves^30^. Importantly, the level of intelligence that children ascribe to robots is positively correlated with children’s depth of understanding the programming that enables the robot to function^30^. To assess how children’s affective perception of robot forms, Woods^31^ gathered ratings of pictures of 40 general purpose robots from 159 children (aged 9–11 years) and found evidence that children perceive humanoid and zoomorphic robots, as well as bright-coloured robots with caricatured faces, as friendlier and more likely to have the capacity for emotions, relative to mechanoid robots. Woods reported that children rated the robots they categorised as human-like, relative to human-machine-like, as more aggressive and angrier. Woods interpreted these findings to mean that in many cases, children may perceive humanoid robots as unsettling and may experience the *uncanny valley effect*, i.e., the sense of unease a person experiences when a non-human entity (e.g., robot) closely, but not fully, resembles a living human^32^. More recent findings suggest that young children may not experience the uncanny valley effect, as it seems to emerge around 9 years of age^33^. Thus, we know that robots that are too human-like are unlikely to be seen as suitable reading companions, yet the specific combination(s) of feature that cause children to perceive robots this way is not yet established.

Returning to co-design, an early study by Lin et al.^34^ found that children (aged 8 + years) designed a library-service robot as a child-size humanoid with machine- (e.g., screens, metal) and toy-like features. Recent studies report similar mixtures of humanoid and machine elements for classroom robots, though the robots were mainly described to be larger than children, have a machine-like voice and express few emotions^21^. In the context of peer-robots, children also designed their robots to be child-size or smaller and to show varying emotional expressions^23,35^. Sanoubari et al.’s^35^ co-design study revealed that children aged 8–12 years preferred human-like features such as a face and desired personalised accessories, decorations, and clothes for their robots. These studies lay foundations upon which a co-design study can explore the ideal features of a reading-specific robot companion.

To date, two co-design studies have explored users’ visions for robots that support reading^25,36^. Cagiltay et al.^25^ involved parents and children aged 10–12 years in the design of a home-based reading robot. The authors found that the robot should take a peer-role and assist with reading, homework, practicing musical instruments, and cooking. Additionally, parents expressed concerns regarding confidentiality of individual’s interactions with the robot and familial interactions, more generally.

Chilufya et al.^36^ engaged teachers and children aged 10–11 years separately to contribute to the design of a robot to stimulate reading motivation. The authors collected design concepts through workshops and distilled the concepts into 10 roles (e.g., buddy, librarian, gamer, etc.). From these roles, a single design was refined for the ‘BookBot’. BookBot’s child-defined competences included the ability to engage in discussions about the book plots and characters to foster shared reading experiences and enjoyment. BookBot was also designed with a personalised book-recommendation function, to encourage continued reading.

These co-design studies highlight the importance of robots’ being customisable in their appearance, behaviours, and content recommendations to suit individual children. Despite the focus on robots as reading companions in the later two studies, the children’s reading levels were not taken into consideration. Readers of all levels appreciated customised reading robot companions, but the specific features beyond ‘a non-judgemental presence’ that would suit children with poor reading remain poorly defined.

### Current study

In this pre-registered study, we aimed to gain deeper insight into features that could meaningfully enhance children’s experiences with robotic reading companions, as well as the reasons that specific features may be important. To do so, we took a mixed-method approach combining a participatory co-design approach with semistructured interviews that were analysed according to Braun & Clarke’s^37,38^ thematic analysis framework to build upon our research group’s mixed-method study with existing robots^15^. As described in more detail in Sect. 1.2, a limitation of Caruana et al.^15^ is that each of the robots had a pre-set appearance and was programmed with specific responses that impacted children’s perceptions. To overcome this limitation, in the present study, we take a co-design approach, thereby creating the opportunity for children to describe behaviours they would like to experience or believe to be beneficial to reading experiences, unbiased by specific implementations of the technology at present. We expect the findings from this co-design study to inform future studies examining robot-guided reading interventions for school-aged children.

Whilst previous work has included adult and child stakeholders within single studies, we have opted to focus on children’s perceptions to maximise the richness of insights relevant to the recipients of the reading support. Given that robotic reading companions’ non-judgemental presence may be particularly valuable for children with reading difficulties and reading anxiety, we also conducted the same co-design activities with a case series of children with a history of reading difficulty, who are likely to experience anxiety about reading^39^. Anxiety about reading and avoidance of reading are, in part, due to a fear of being judged negatively when reading errors are made^40^. We thus collected perspectives from children with lived experience of reading difficulty to determine whether any nuances in the needs and expectations of children with poor reading skills regarding reading robot companions that may differ from the needs and expectations of children with typical reading skills.

## Methods

### Participants

#### Children with typical reading

As per our preregistration (https://osf.io/dmbta), the community sample included 31 children (17 female) between the ages of 5–9 (*M* = 7.03, *SD* = 1.37 years) with typical reading abilities. The age range of 5–9 years spans children’s introduction to formal reading instruction and also covers the time when reading difficulties or delays are most likely to first become apparent. As such, this is also when children would benefit from reading interventions. Children with typical reading scored < 1 standard deviation below or higher than the Australian age-specific norms^41^ on both subscales of the Test of Word Reading Efficiency 2 (TOWRE2; measure described in Sect. 2.2.1). In other words, children with typical reading are those who fall within the normative range of performance on our selected measures of reading efficiency. While we acknowledge that reading skill is multifaceted, and can be operationalised in many ways, we focused on reading fluency in using the TOWRE-2 because it is one of the most widely used objective measures of reading skill, is practical to administer, and because fluency is one of the aspects of reading skill that is most likely to associate with children’s experiences of anxiety when reading in front of others. This follows in that reading disfluently in front of others is an obvious behavioural marker of reading ability, whereas poor comprehension or hyperlexic reading, when reading to others, is not. One child was excluded from analysis due to difficulty cooperating during the interview and design task.

Most participants’ (90.32%) first language was English. The study was advertised via social media pages, as well as posters and flyers, distributed throughout local libraries and parent clubs within the Northern and Northwest suburbs of Sydney, Australia. Socioeconomic status information is not available for our participants. Nonetheless, it is likely that most children came from average or above-average income households given the location of the university and clinic, and the unsubsidised cost of attending regular reading remediation.

#### Children with poor reading

Extending beyond our preregistration, we conducted a case series of children with poor reading. We recruited five additional children (3 female) aged 7–10 (*M* = 8.60, *SD* = 1.40 years). Of these children, four (2 female) were regularly attending one-on-one reading remediation sessions at Macquarie University Reading Clinic and had undergone extensive diagnostic assessments to identify reading difficulties warranting remediation. An additional child with reading difficulty (PR03; female) was recruited from the wider community. This child had been identified as having reading difficulty with dyscalculia and ADHD by a pediatric clinic specializing in neurodevelopment and behavioural disorders. All five children scored > 1 standard deviation below the age-specific average on at least one of the three subscales of the Castles and Coltheart reading test (CC2; individual scores in Table 1, scale description in Supplementary Materials A), confirming marked difficulty in at least one domain of reading. The domains include decoding and sight reading of whole words. All children spoke English as first language.

#### Ethics statement

Each session lasted 60 min and families received $20 AUD to thank them for their participation. All the methods were carried out in accordance with relevant guidelines and regulations stipulated in the approval granted by the Macquarie University Ethics Committee (reference: 52023990346922).

### Measures

To characterise our samples and contextualise the perspectives offered in our qualitative analysis, we collected measures of reading ability and anxiety. Children with poor reading and their parents completed additional measures to assess whether children met the inclusion criteria for the case series. Individual and group level scores were computed using R and R studio (data and code available on OSF; https://osf.io/395gh/).

#### Reading fluency

The (TOWRE-2) assesses children’s reading fluency^42^. We administered two subtests (from form A): Sight Word and Phonemic Decoding. The Sight Word subtest assesses a child’s ability to fluently recognise whole words based on the orthographic representation using a series of regular and irregular words that become progressively more difficult. The Phonemic Decoding subtest assesses the child’s ability to decode words that can be pronounced in English, using grapheme-correspondence rules (i.e., by ‘sounding out’), but do not exist in the English dictionary. For both substests, the number of words accurately read aloud by the reader in 45 s is counted, converted to a standardised score, and matched to Australian age norms^41^. Scores for children aged 5 years were standardised according to standardisations for the TOWRE-2’s youngest age group (6 years).

#### Reading anxiety

The Reading Anxiety Test for Children – Parent Report (RAT-CP) was included in the parent questionnaire in this study^43^. The RAT-CP measure includes 48 items of statements describing a child’s behaviour towards reading (e.g., “*My child worries that s/he is not a good reader”*). Parents were asked to respond to each of the statements by rating how often their child exhibit such behaviours from 0 = ‘Never/I don’t understand” to 3 = “Always”. Total raw scores obtained were included in the analysis. Scores from 0–13 reflect low anxiety, scores 14–28 reflect elvated reading anxiety, and scores above 29 reflect highly elevated reading anxiety (i.e., 99th percentile).

#### Additional measures in case series of children with poor reading

The parents of children in the case series of children with poor reading completed the Spence Children’s Anxiety Scale (SCAS-P), The Negative Evaluations Test parent report (NeST-P), and The Conners-3 – P (short). The NEST-P measures children’s perceptions that they have received negative evaluations from other people about their reading. The Conners-3 measures inattention. To assess reading accuracy, children completed the Castles and Coltheart 2 test (CC2). See Supplementary Materials A for full descriptions of these measures.

### Procedure

Sessions were conducted at Macquarie University by two female researchers (one per session) following a pre-defined protocol (available on OSF: https://osf.io/395gh/). First, informed consent was obtained from both the parent and child. Parents completed questionnaires about their children (i.e., demographic information, hobbies, and a reading anxiety measure). Seven parents of children with typical reading opted to accompany their child during the session, as did three parents of children with poor reading.

During the session, the experimenter and child sat side-by-side at a small table. Parents who accompanied children sat approximately 2 m behind the experimenter and child, in the room but out of eyesight. Reading assessments were completed (see Sect. 2.3) to obtain individual scores of reading ability and to prime children with a recent reading experience to help contextualise their thinking about how a robot could support them during reading.

#### Participatory design

Next, the experimenter asked the child questions about their understanding and prior experience with robots. Subsequently, to initiate the co-design procedure, children were given coloured pencils and a blank sheet of paper and were asked to draw a “*robot reading buddy”* that could be used to help children with reading. When the child had finished their drawing, the experimenter proceeded to ask questions to explore the design elements of the robot’s physical form, functional capabilities, and personality with the child. Questions included: *“Could you tell me a bit more about what it looks like and what it can do?”*, *“What is it made from?”*, *“Why do you think your robot would make a good reading buddy robot?”*, *“Does your robot have any other parts or features that you did not draw here?”*, *“How does this robot communicate with you?”*, *“How do you feel when you spend time with this robot?”*, and were followed up with prompt to encourage expression of further or information: *“Tell me more about that”*, *“Why (not)?”*. Interviews were audio-recorded and transcribed verbatim for both the qualitative thematic analysis. A full list of potential prompts can be found in the complete protocol on OSF and in Supplementary Materials B. Raw transcripts are available on OSF (https://osf.io/395gh/). Children were invited to opt in for their robot designs to be included in an online gallery (https://www.soba-lab.com/soba-kids).

#### Impressions of existing robots

Upon completing the co-design task, children viewed existing robots, either physically in the room, in videos, and/or in static pictures. While children’s verbal responses to these experiences were included in the thematic analysis, we focus primarily on insights gained during the design process. Quantitative perceptions of existing robots (i.e., ratings) were elicited using verbal questions that were supplemented with visual aids. Ratings are reported in the Supplementary Materials C.

##### Children with typical reading

 Once the child had completed their initial design, children with typical reading viewed a video of a humanoid NAO robot reading a short story to the zoomorphic MiRo and mechanical Cozmo robots. The purpose of these videos was to showcase examples of social robots that differ in their appearance and functional ability to elicit more insights from children who may have struggled with the unconstrained task of drawing a reading robot companion.

##### Children with poor reading

 We implemented a more in-depth protocol for interviewing children with poor reading to capture much of the same information previously gathered from children with typical reading and reported in Caruana et al.^15^. As such, following the unconstrained design task, children with poor reading were introduced to three physically present, but inactive robots: the NAO, MiRo, and Cozmo robots. Children ranked the robots from most to least preferred, then viewed videos of each robot rolling or dropping a ball off the edge of a table and respondingly expressively to the ball falling. This allowed us to record children’s perceptions based on the appearance and functional capabilities of robots separately. After each video, children rated the *kindness* and *intelligence* of each robot from ‘very’ to ‘not at all’ (using a visual analog scale from 1 to 3, with visual aids instead of text anchors for scales). This was then followed by a second ranking of robots by preference. Finally, children viewed a video (*n* = 3) of an out-of-frame person reading a book to each robot, where the robot made appropriate emotional responses to plot points. After each video, children were asked again to rate the *kindness* and *intelligence* of the robot in the video.

### Qualitative thematic analysis

The aim of the qualitative thematic analysis was to capture the features that children want and expect from a robotic reading companion. Transcription of interviews was performed via otter.ai, after which transcripts were reviewed and inaccuracies corrected by HC. Transcripts were analysed following a reflexive thematic analysis approach^37,38^, wherein HC generated initial codes and themes using NVivo (Version 20). Initial thematic analysis was conducted by HC and NC who reviewed transcripts and identified codes and themes through an inductive, iterative and collaborative process for each sample separately. Next, the codes, thematic structure, and extracted evidence was reviewed and discussed by all authors. Full transcripts and summaries of codes with children’s statements are available on OSF (https://osf.io/395gh/).

## Results

### Reading scores

#### Children with typical reading

**Fig. 1:**
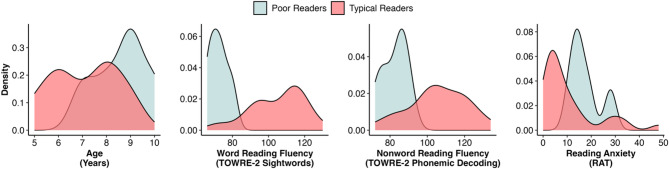
Distributions of children’s ages, as well as reading and reading anxiety scores, for typical (*n* = 30) and children with poor reading (*n* = 5).

 Based normative data for the TOWRE-2, children had above average reading fluency for both sight words (*M* = 104.65, *SD* = 14.42) and phonemic decoding (*M* = 105.45, *SD* = 5.13). The group level mean for reading anxiety scores on the RAT-CP was generally low (*M* = 10.39, *SD* = 11.65; Fig. 1).

#### Children with poor reading

 Children with history of reading difficulties scored within the expected range for their age, and numerically lower than children with typical reading, for both sight words (*M* = 72.4, *SD* = 5.41) and phonemic decoding (*M* = 82.4, *SD* = 6.80). Reading anxiety for the RAT-CP scores were greater than in children with typical reading with reduced variability (*M* = 17.4, *SD* = 6.58; Fig. 1). As the case series was exploratory and not part of our preregistrations, we did not preregister or conduct any statistical comparisons between children with typical and poor reading. All measures collected as part of case series are reported Table 1.

**Table 1:** For TOWRE-2 and CC2 scores, ↑ indicates score > 1 standard deviation above age-specific average and ↓ indicates scores > 1 standard deviation below age-specific average.

Demographic/measures	Score type	PR01	PR02	PR03	PR04	PR05
Age/gender	–	9/M	7/F	10/F	8/F	9/M
Diagnosis	–	Dyslexia	None	ADHD/Discalculia	Dyslexia	ADHD
TOWRE—sight word	Standardised score	75	66	80	69	72
TOWRE—phonemic decoding	Standardised score	73	90 ↑	86 ↑	85 ↑	78
CC2—regular words	Z-score	− 2.33 ↓	− 1.29 ↓	− 0.22	− 0.78	− 1.87 ↓
CC2—irregular words	Z-score	− 2.03 ↓	− 1.65 ↓	0.63	− 2.15 ↓	− 1.67 ↓
CC2—nonwords	Z-score	− 2.03 ↓	− 0.65	− 1.35 ↓	− 0.38	− 1.87 ↓
RAT-P—reading anxiety	Percentile	11	19	14	28**	15
SCAS- P—total	T-score	51	40	63**	50	47
SCAS- P—generalised anxiety	T-score	57	40	65**	52	48
SCAS- P—social phobia	T-score	50	41	43	51	55
NeST – P—negative evaluation	Total score	11	19	14	28	15
Conners 3 (short)—inattention	T-score	65	52	87**	73	56

Of the CC2 subscales, regular words and nonwords measure decoding, while irregular words measure whole word reading. For the SCAS and Conners 3, ** indicates elevated scores compared to normative data.

### Robot designs

#### Children with typical reading

**Fig. 2:**
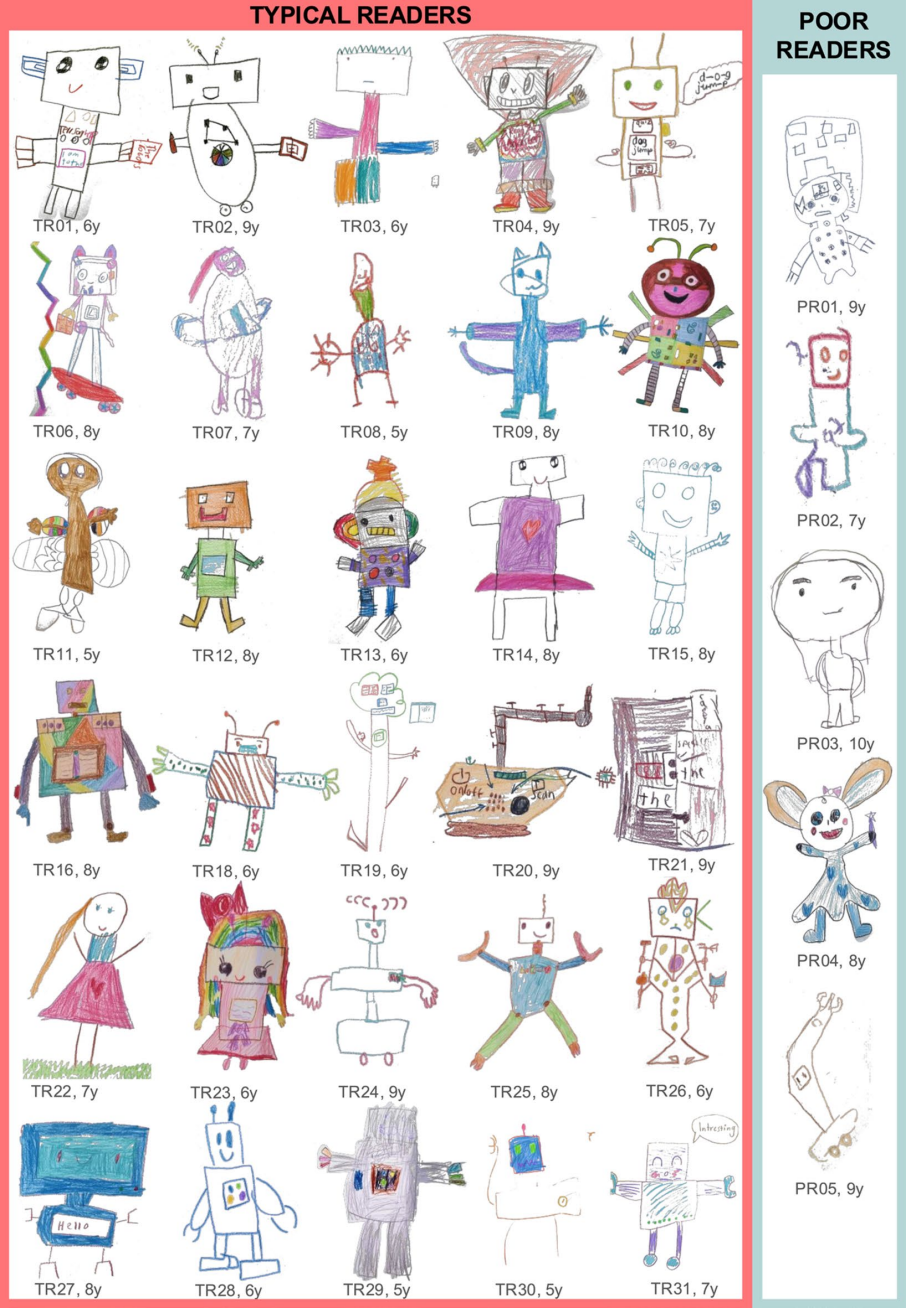
Robot designs illustrated by children with typical reading (IDs beginning with TR) and children with poor reading (ID beginning with PR) during the participatory design part of the session. ID are followed by child’s age in years.

 Children’s illustrated robot designs were diverse, but primarily featured humanoid reading robots (*n* = 25). These designs combined human-like faces, bodies/torsos, and arms, with mechanical features, such as screens and buttons. The remainder resembled zoomorphic agents (e.g., cats; *n* = 2), mechanistic gadgets (*n* = 2), and in one case, an animate tree-like companion (*n* = 1). Robot designs are presented in Fig. 2.

#### Children with poor reading

 Robot designs by children with poor reading depicted humanoid agents (*n* = 3), as well as a zoomorphic (*n* = 1) and a mechanoid agents (*n* = 1; Fig. 2). Robot designs are included in Fig. 2.

### Thematic analysis

**Fig. 3:**
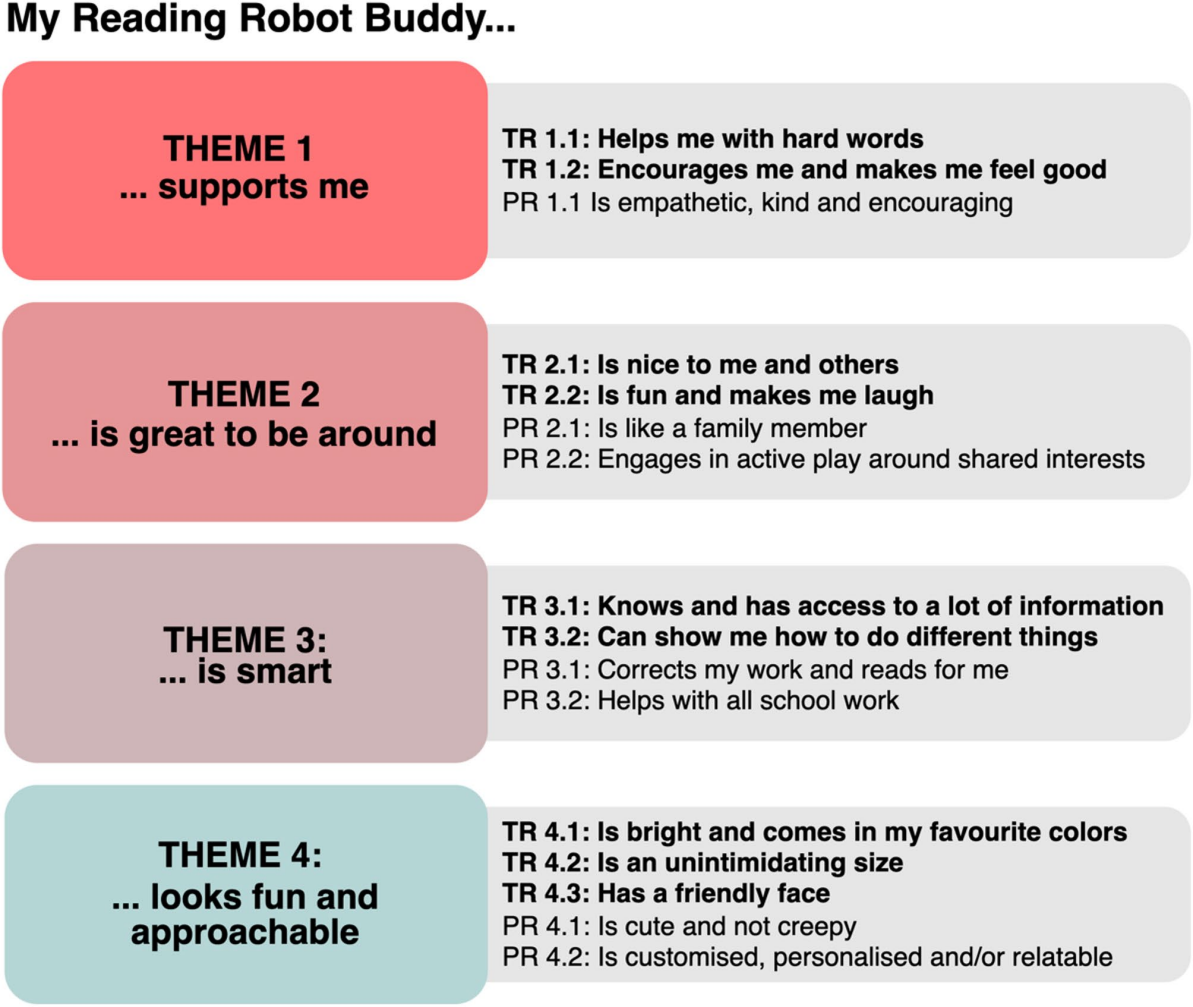
Summary of themes and subthemes. The overarching themes are the same for typical (TR) and poor (PR) readers, whereas the subthemes are recorded separately.

A comprehensive summary of themes and subthemes is presented in Fig. 3. Typical (TR) and poor (PR) readers interviews were analysed separately. Children’s statements are accompanied by a reference code made up of the child ID, age in years, and RAT-CP score to provide context regarding reading anxiety (presented in the following format: [ID, age, RAT-CP score]).

#### Theme 1. My robot reading buddy supports me

 Children with typical reading’s robots were designed to provide reading-specific support (**subtheme TR 1.1**) by helping the child to decode words. TR11 [5y, 48] said “*if you’re really stuck or he can help you sound them out”*. Children also proposed general learning support while reading. TR05 [7y, 2] offered “*it’s got two screens*,* and one of thems’ a book and the other one … like a testing page… and then it chooses a quiz”.* TR20 [9y, 5] explained “*maybe the robot will suggest like a little trick or like a little song to help remember”*. TR06 [8y, 3] suggested “*he does this like four times so then they can like kinda memorise it”*. Similarly, children with typical reading were also explicit that emotional support would build reading confidence (**subtheme TR 1.2**). One such statement was *“if they get it wrong*,* they don’t correct them… they get more confident and then they can start reading with humans”* [TR12, 8y, 5]. Children with poor reading designed empathetic and kind robots that encouraged reading engagement (**subtheme PR 1.1**). PR03 [10y, 14] specified *“it just won’t ever be angry or sad. Unless you’re sad. It’ll be sad if it will be sad if you’re sad and try to cheer you up”.* PR03 [10y, 14] explained they would *“say*,*’I am going to be reading’ and then like it’ll cheer you on”*.

#### Theme 2. My robot reading buddy is great to be around

 Children with typical reading expressed that their robot is friendly and embodies prosocial behaviours (**subtheme TR 2.1**). For example, TR18 [6y, 0] described their robot as inclusive: *“he wouldn’t leave his friends out if they want to play game together”*, while TR06 [8y, 3] mentioned their robot’s patience: “*she would be very kind and not rush you”*. Children with typical and poor reading designed their robots to provide entertainment (**subthemes TR 2.2** and **PR 2.1**). Children’s choices of entertainment involved active play such as dancing, soccer, boardgames, making funny faces, and telling jokes to induce laughter. Typical and children with poor reading alike described their robot as a conversationalist. PR01 [9y, 11] said *“he can talk about anything as long as you want to talk about it”*. Some children with poor reading ascribed sibling-like or family-member roles to their robot (**subtheme PR 2.2**). One example expressed by PR04 [8y, 28] was *“I would love a big sister*,* who is older than me and taller than me and she’d be like my big sister”*.

#### Theme 3. My robot reading buddy is smart

 Children with typical reading desired that their robot be highly knowledgeable across various topics (**subtheme TR 3.1**). TR02 [9y, 8] explained *“it can teach you Maths*,* English*,* History*,* so it could just technically be your teacher if you’re doing homeschooling”*. Some children with typical reading described how their robot access the information on the internet: “*his brain is connected to the internet*,* he’d be good at most things learning-wise”* [TR21, 9y, 7] and made their robot multilingual: *“it can understand any language because it might want to help kids from another country”* [TR06, 8y, 3]. Importantly, children with typical reading expect their robot share their knowledge in an informal teaching capacity (**subtheme TR 3.2**). TR04 [9y, 1] stated *“He’s like a friend*,* not really a teacher. But he’s like*,* he can help like the kids because he’s like*,* really smart”*. Much like children with typical reading, children with poor reading designed their robots to help with a variety of school subjects (**subtheme PR 3.1**). PR04 [8y, 24] listed *“Maths*,* English*,* everything mum helps me with. Except for like tests*,* like reading questions”*. Children with poor reading also suggested that robots should provide constructive learning support (**subtheme PR 3.2**). PR03 [10y, 14] explained *“it wouldn’t just tell you the answer*,* it would like help you to like figure it out”*.

#### Theme 4. My robot reading buddy looks fun and approachable

 Children with typical and poor reading emphasised the importance of bright colours (**subthemes TR 4.1** and **PR 4.2**) and aligning the robot’s colour to a child’s favourite colour. TR19 [6y, 3] said “*because if there’s only one colour*,* some people don’t really like only that colour”*, a sentiment echoed by P001 [9y, 11]: *“because if it was just the same old colour it would just get boring… and you don’t want to buy if you can just switch colour*,* it switches it’s colour”.* With respect to robot size (**subtheme TR 4.2**), children from both groups proposed compact robots, often describing child-sized or smaller designs. Children with typical reading offered considerations for enhancing approachability: “*if he’s very big then he might be giant*…*scary… and mean* ” [TR03, 6y, 1], portability: “*then they could take her or him to different places… it’s easier to pack*” [TR06, 8y, 3], and reading ergonomics: “*the kids can like be at the same level as him- at the same height… and that they don’t have to stretch their neck up to see so high*” [TR27, 9y, 5]. Children with typical reading described the importance of friendly (non-scary) smiles (**subthemes TR 4.3** and **PR 4.1**). TR15 [8y, 11] explained *“the smile might help them to be happy and be more confident in reading*”. Some children with poor reading focused friendly facial features *“so*,* [they] wouldn’t get creeped out as much”* [PR03, 10y, 14]. Customisation of robots to make them relatable was common among children with poor reading (**subtheme PR 4.2**). For example, children with poor reading often described that their robot’s name was derived from a close social connection, perhaps suggesting that children see the robot as a proxy for supportive companionship. PR02 [7y, 19] said *“Winston*,* that’s my dog’s name”* and PR01 [9y, 11] said *“KLM.*. *it stands for three of my friends”*.

## Discussion

Our thematic analyses offer insight into design features that may help optimise social robots for assisting children in learning to read, as well as providing social companionship more generally. While discussing the robot they designed, children expressed that reading robot buddies should: (1) be supportive on reading-specific and emotional levels; (2) provide enjoyable companionship comparable to friends and family members; (3) behave ‘intelligently’ by providing information across many topics; and (4) have a fun and approachable appearance. Though interviews with children with typical and poor reading were analysed separately, we found the main themes to be the same. The subthemes, however, shed light on additional nuances.

### Reading robots that provide reading and general emotional support

Most children with typical reading designed their robot to help readers ‘sound’ or ‘spell’ out difficult or new words. A few children with typical reading made a connection between reading and writing and wanted the robot to be able to assist with writing or use writing to support reading. Other supplementary functions suggested by children with typical reading included pointing with a finger at misread words, generating tests and quizzes, tips, and initiating memorisation games. These calls for corrective feedback align with and extend on children’s suggestions in our previous work^15^. Some children with typical reading suggested that their robot would update their parents on progress, which aligns with existing research that robotic reading companions can integrate into a family as a whole^25^.

Children with typical reading described how their robots would build reading confidence through emotional support and encouragement. Children with poor reading also highlighted their robot’s ability to express emotions and empathy, often emphasising that their robot was kind and encouraging. Children’s statements resonate with recent findings that negative evaluations of reading performance and reading axiety are associated with poor reading and may underpin reading avoidance^44,45^. Moreover, amassing evidence suggests that reading with a robotic companion, may offer a more comfortable, non-judgemental, environment for anxious children than conventional reading with other people^15–17,46^. Finally, children with typical reading emphasised that their robots would recognise children’s emotions during reading and beyond, respond appropriately to distress by providing more general emotional support. Indeed, this has been identified as a critical component of positive social interactions between children and educational robots^46^. However, while some initial evidence suggests that interactions with social robots can mitigate anxiety^47,48^, additional research is warranted. Additional co-designed research should examine how robot form and function can maximise these forms of emotional support and give rise to optimal psychosocial conditions for children to engage in reading and other learning activities. Given the current limitations of robot interactions mediated by artificial intelligence (AI), ethical concerns remain as to whether robots can accurately detect and respond to children’s emotional states in a reliable, safe, and autonomous way^46,49^.

## Conclusions

Given the opportunity to design their own reading robot companions, children with and without reading difficulties designed robots that could provide not only reading-specific support, but also emotional support during reading activities and in everyday life. Children’s descriptions of their robots’ behaviour reflected prosocial behaviours that are beneficial in friendships and sibling relationships. Robots were expected to be very knowledgeable and have access to information about many topics, as well as to be colourful and continuously personalisable. These qualitative findings highlight that robotic reading companions stand the greatest chance of benefiting young readers, particularly those with reading difficulties or anxiety, if the robots are tailored to the readers’ needs and expectations.

## Supplementary Information

Below is the link to the electronic supplementary material.

Supplementary Material 1

## Data Availability

The datasets generated and analysed during the current study are available in the Open Science Framework repository, along with associated code and other supplementary materials (https://osf.io/395gh/).
