# Supplementary material for: Children with and without reading difficulty value robot reading companions that are smart, supportive, and personalised
Source: Sci Rep. 2025 Oct 1;15:34178. doi: 10.1038/s41598-025-15341-w (PMC12488995; doi:10.1038/s41598-025-15341-w)
Supplement: Supplementary file 1 — Supplementary Material 1 [file 41598_2025_15341_MOESM1_ESM.pdf]

**Supplementary Material:** Moffat et al. – "It'll cheer you on!" Children with and without reading difficulty value robot reading companions that are smart, supportive, and personal

## A. Additional reading measures collected from poor readers.

**Reading accuracy.** The Castles and Coltheart 2 (CC2) test was administered to characterise reading accuracy (Castles et al., 2009). The CC2 examines whether there is a deficit in either the non-lexical route (reading irregular words that do not follow grapheme-phoneme correspondence rules) and the lexical route (reading non-words by following grapheme-phoneme correspondence rules) or the non-lexical route. This test involves 40 regular words, 40 irregular words and 40 non-words. These are presented one at a time in a pseudo-randomised order that increases in difficulty.

**General and Social Anxiety.** The parent report of the Spence Children's Anxiety Scale (SCAS-P) was used as an overall measure for anxiety and phobias (Spence, 1997). Parents rate from 1-3 (1=sometimes, 2=often, 3=always) the frequency of an anxiety symptom (e.g. "*My child worries that something bad will happen to him/her*").

**Negative Evaluation.** The *Negative Evaluations Test* parent report (NeST-P) measures children's perceptions that they have received negative evaluations from other about their reading (McArthur & Francis, 2022). The parent/guardian completes this scale of 9 items of statements (e.g. "*My child is picked on because of their reading*"). Responses are either "true" or "not true" to statements of where the child has been receiving feedback for their reading ability, such as their peers or teachers.

**Inattention.** The *Conners-3 – P (short*) is a shorter version of a parent report Scale that measures child's inattention and hyperactivity (Conners et al., 2011). There are 44-items relating to the child's behaviour within the last month (e.g. "*Has trouble getting started on tasks or projects*"). Likert ratings are from 0-3, with 0 = never/not true to 3 = often/true.

### Group level scores (poor readers)

| Measures | *M* | *SD* |
|---|---|---|
| RAT-P – Reading Anxiety | 17.4 | 6.58 |
| SCAS- P – Generalised Anxiety | 3.6 | 2.07 |
| SCAS- P – Social Phobia | 3 | 1.414 |
| NeST – Negative Evaluation | -1.374 | 1.141 |
| CONNERS 3 (Short) - Inattention | 66.6 | 14.01 |

Castles, A., Coltheart, M., Larsen, L., Jones, P., Saunders, S., & McArthur, G. (2009). Assessing the basic components of reading: A revision of the Castles and Coltheart test with new norms. *Australian Journal of Learning Difficulties*, *14*(1), 67–88. https://doi.org/10.1080/19404150902783435

Coltheart, M., Rastle, K., Perry, C., Langdon, R., & Ziegler, J. (2001). DRC: A dual route cascaded model of visual word recognition and reading aloud. *Psychological Review*, *108*(1), 204–256. https://doi.org/10.1037/0033-295X.108.1.204

Conners, C. K., Pitkanen, J., & Rzepa, S. R. (2011). Conners 3rd Edition (Conners 3; Conners 2008). In J. S. Kreutzer, J. DeLuca, & B. Caplan (Eds.), *Encyclopedia of Clinical Neuropsychology*. Springer New York. https://doi.org/10.1007/978-0-387-79948-3

McArthur, G., & Francis, D. A. (2022). *The Negative Evaluations Test (NEsT)*. https://www.motif.org.au/nest

Spence, S. (1997). Spence Children's Anxiety Scale (SCAS). *Journal of Anxiety Disorders*. https://doi.org/10.1037/t10518-000

## B. Prompts during design task

Thank you for doing that. Describe your reading buddy robot to me? What have you created? Anything else you want to tell me about your robot?

**General prompts**

- Could you tell me a bit more about what it looks like and what it can do?
  - What is this?
  - What does this do?
  - Why have you given it…
  - And why is it important that it does / has that?
- Can you point out some things that make this robot special/unique/different to other robots?
- Why do you think your robot would make a good reading buddy robot? What can it do to help children who find reading hard or scary?
- Let's give your robot a name. What would you call your robot?

**Explore physical features (additional probes to help children structure thoughts supplied)**

- What is it made from?
- How would it feel to the touch?
  - *Would it be soft and cuddly or hard and shiny?*
  - *Why do you like that?*
- I see you've made it XXX colour
  - *Why did you choose those colours?*
  - *Could it come in other colours?*
- How big would it be?
  - *Would it be small enough to put in your pocket or bigger than you or somewhere in between.*
  - *Why would you like it to be that size?*
- Does your robot have any other parts or features that you did not draw here?
  - *What do they help it do?*
- Does this robot have a face? (or, I can see this robot has a face…)
  - *Why do you want it to have a face?*
  - *What sort of face does it have?*
  - *Does any part of its face move?*
  - *Why is that important?*

**Explore robot capabilities**

- How does this robot communicate with you?
  - *Can this robot talk to you?*
  - *How does it do that?*
  - *Does it understand and speak English?*
  - *What does it talk about?*
  - *What things does this robot know about?*
- What does this robot do to help with reading?
  - Probe: words, actions, movements, expressions
  - Probes: why is that important, how would they do that
  - Can this robot read?
- What else can this robot do?
  - *What things is this robot good at?*

- *What things is this robot **not** good at?*

- Can this robot move?
  - *How does it move?*
  - *What things can it do if it moves?*
- Other than helping with reading, can this robot do anything else with you?
  - *What other things would you like this robot to be able to do?*

**Explore robot personality features**

- How do you feel when you spend time with this robot?
- What words could you use to describe how this robot acts towards you?
- Do you think this robot might be naughty or nice?
  - *Tell me more about that.*
- Do you think you could tell this robot a secret?
  - *Why/ why not?*
- Do you think this robot might be friendly or scary?
  - *How do you know?*
  - *What does it do that is…?*
- Do you think this robot is smart?
  - *How do you know if it is…?*
- Is this robot like your friend or your teacher or something else? *(rotate order of friend/teacher)*
  - *What does it do that makes you think it's like your…?*
- Is there anything else you want to tell me about what makes this robot a special reading buddy?

## C. Impressions of existing robots:

Poor readers (n = 5) complete an activity modelled very closely on Caruana et al. (2023) protocol. The figures below are provided to enable comparison with the data presented in Caruana et al. (2023). Code available on OSF.

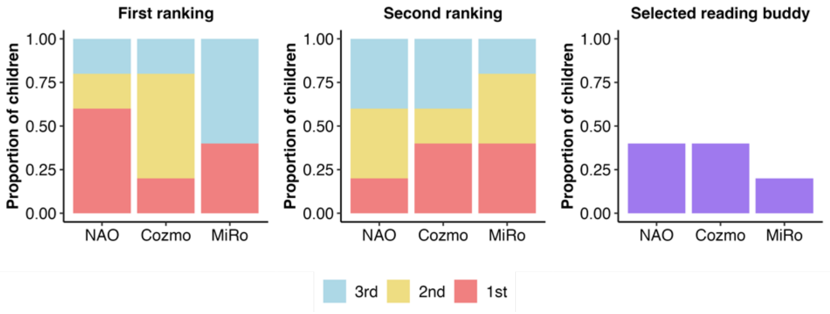


**Figure A.** Rankings of robots after seeing robots standing still in the lab (left), after watching a video of each robot rolling a ball off the table and responsing appropriately (middle), and the proportion of children who selected each robot as their reading buddy (right).

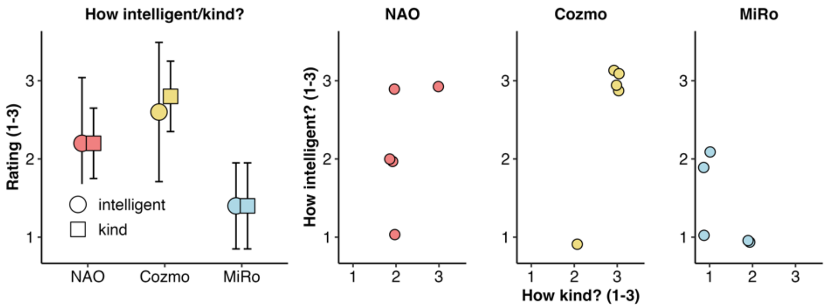


**Figure B.** After reading to the robot of their choice, the children rated all three robots' intelligence and kindness. This figure shows ratings of robot's intelligence and kindness (left), and the three pannels to the right show intelligence ratings as a function of kindness ratings per robot.

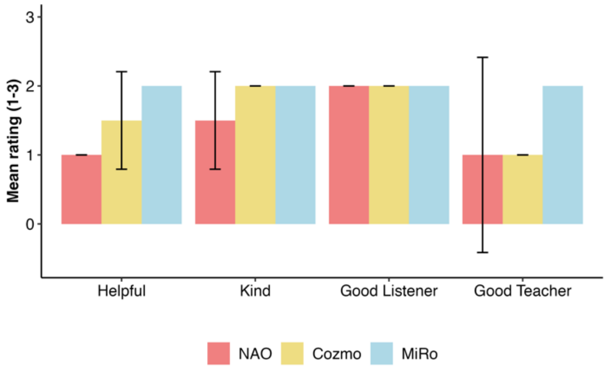


**Figure C.** Subsequently, each child rated the robot that they read with on on four dimensions: How helpful and kind the robots is, as well as how good of a listener and teacher the robot is. Here, 2 children rated NAO, 2 children rated Cozmo, and 1 child rated MiRo.

Caruana, N., Moffat, R., Miguel-Blanco, A., & Cross, E. S. (2023). Perceptions of intelligence & sentience shape children's interactions with robot reading companions. *Scientific Reports*, *13*(1), 7341. https://doi.org/10.1038/s41598-023-32104-7
